# Adult users of a digital binge-eating intervention wanted adaptations for type 2 diabetes: Qualitative study of post-intervention preferences

**DOI:** 10.21203/rs.3.rs-10019310/v1

**Published:** 2026-07-08

**Authors:** Isabel R. Rooper, Alyssa M. Vela, Rebecca L. Flynn, Rosa Saavedra, Thomas Vorisek, Nathan Chan, Graham Miller, Jennifer E. Wildes, Andrea K. Graham

**Affiliations:** Northwestern University Feinberg School of Medicine; Northwestern University Feinberg School of Medicine; Northwestern University Feinberg School of Medicine; Northwestern University Feinberg School of Medicine; Northwestern University Feinberg School of Medicine; Northwestern University Feinberg School of Medicine; Northwestern University Feinberg School of Medicine; University of Chicago; Northwestern University Feinberg School of Medicine

**Keywords:** Binge eating, diabetes mellitus, diabetes care, feeding and eating disorders, user-centered design, qualitative research

## Abstract

Purpose: Binge eating, defined as eating a large amount of food while feeling a loss of control, impacts up to 25% of adults with type 2 diabetes mellitus (T2DM). Although binge eating worsens physical health and mental health in adults with diabetes, binge-eating interventions have rarely been adapted for diabetes. To address this gap, we asked adults with T2DM and binge eating who completed a digital binge-eating intervention (FoodSteps) about challenges they experience and opportunities to adapt FoodSteps for diabetes. Methods: Participants were drawn from two trials testing FoodSteps. Eligible participants were adults (≥18 years) with T2DM and recurrent binge eating (≥12 episodes in the past 3 months). Post-intervention interview data were analyzed using methods from thematic analysis. Results: Forty-two participants were included: 74% self-identified as female; 14% as Hispanic, 14% as non-Hispanic and another race, 33% as non-Hispanic Black, and 38% as non-Hispanic White. Participants described challenges managing T2DM due to knowledge barriers, cognitive/psychological barriers, physiological barriers, and external/structural barriers. Participants suggested adaptations to address these barriers, including psychoeducation, reminders, glucose tracking features, and increased medical support and social support. Participants said that individuals recently diagnosed with T2DM may benefit most from adapted intervention. Conclusions: Results indicate opportunities to adapt the design and delivery of binge-eating intervention for diabetes, though these design ideas must be validated with new users. This research has implications for diabetes care clinicians, who may benefit from addressing patients’ eating behaviors. Future work will design adapted intervention features with clinicians and adults with diabetes.

## Introduction

Type 2 diabetes mellitus (T2DM) impacts 12% of adults in the United States [1], with forecasting models for 2030 predicting that T2DM prevalence rates will continue rising [2]. T2DM disproportionately burdens low-income and minoritized individuals, particularly those who identify as American Indian/Alaska Native, Hispanic, or Black [3], making T2DM an important target to improve health equity. Individuals with T2DM commonly experience binge-eating disorder and binge-eating behaviors, which involve eating a large amount of food and experiencing a loss of control while eating [4]. Though the scope of co-occurring T2DM and binge eating is large and not fully known, prevalence estimates of binge-eating disorder in adults with T2DM (1.4–25%) [5] are typically higher than in the general population (2–3.5%) [6]. Subclinical binge-eating behaviors are also common, impacting up to 40% of people with T2DM [7]. Evidence on the directionality of the relationship between T2DM and binge eating is mixed, suggesting a bidirectional relationship in which they exacerbate each other [8].

Managing T2DM while experiencing binge eating can worsen individuals’ quality of life [9], physical health, and mental health. Physically, binge-eating behaviors are linked with impaired insulin sensitivity and increased risk of insulin resistance [10]. Binge eating worsens glycemic control and other metabolic markers [11, 12], which can yield microvascular complications such as neuropathy and retinopathy [13]; conversely, reducing binge eating can improve glycemic control [14]. Notably, binge eating can compromise weight management, a key focus of T2DM care [15]. People with T2DM and higher body weight are at elevated risk of developing eating disorders [7], and binge-eating disorder is associated with higher body mass index (BMI) in adults with T2DM [11]. Indeed, presence of a comorbid eating disorder can lead to weight gain [16] and difficulties with weight loss [17, 18]. This may be because the low-calorie, restrictive diets prescribed in T2DM care can exacerbate binge eating [19, 20]. Also, insulin therapy may induce cravings for high-sugar, energy-dense foods [21], which often comprise binge-eating episodes [22]. Regarding mental health, distress is elevated and impairing in adults with T2DM and an eating disorder [17]. Adults with T2DM often experience diabetes stigma and internalized weight stigma, which can increase binge eating [23], and binge eating is also associated with anxiety, guilt, shame, and distress [19, 21, 24, 25]. Feeling guilty about the glycemic effects of binge eating can lead individuals to binge eat as a coping strategy [21], exacerbating a cycle of binge eating and T2DM-related consequences.

Despite the prevalence and impairment of binge-eating behaviors in adults with T2DM, binge-eating interventions have rarely been tested in people with T2DM or adapted for T2DM. Extant efforts have focused on detecting binge eating in diabetes care settings via screening [26] or clinical conversations [27]. This is an important step, but research also must ensure that the ensuing intervention is relevant to adults with T2DM. Prior efforts to do so are reviewed below. Kenardy, Mensch [14] tested a 10-week cognitive behavioral therapy (CBT) group-based intervention for binge eating in women with T2DM; despite initial evidence for sustained reductions in binge eating (compared to non-prescriptive therapy), it was not tested further. Vela, Palmer [28] tested a 12-week CBT guided self-help intervention in 10 women with T2DM and disordered eating. The intervention, which was not adapted for T2DM, showed within-subject reductions in disordered eating behaviors. Lastly, researchers adapted a transdiagnostic guided self-help intervention for eating disorders to create POSE-D, a 12-week digital intervention for adults with T2DM who experience binge eating [29]. POSE-D encompasses a digital tool and seven hour-long sessions with trained professional guides (psychologists, nurses, dietitians). Intervention adaptations for T2DM focused on the human support model, wherein guides offer users individualized support based on their specific T2DM-related challenges. In a pilot study (N = 22), the intervention reduced binge eating and improved T2DM outcomes [30], indicating that guided self-help interventions may be well-suited to address this population’s needs. While digital interventions are often poised for scalability, POSE-D relies on clinical professionals for delivery, which is challenging because there are insufficient clinicians to meet extant needs. The intervention also was designed with input from just three end-users [29], whereas design processes often engage a diversity of perspectives to more fully understand end-users’ needs [31].

Indeed, human-centered design is an approach that uses interactive methods to center the perspectives of target end-users throughout an intervention’s development [32]. The methods can be used to design eating disorder interventions that fit into users’ lives [33]. Our team has engaged over 400 adults with binge eating in human-centered design to create FoodSteps, a 16-week guided self-help digital intervention for binge eating and weight-related behaviors. FoodSteps has achieved high engagement [34] and is poised for scalability as it includes support from non-specialist coaches, aligned with the supportive accountability model for human support in digital interventions [35]. Though past trials of FoodSteps have included adults with T2DM, FoodSteps has not been adapted for T2DM. To learn if and how to adapt FoodSteps, we interviewed participants post-intervention as part of our ongoing human-centered design efforts. Our goals were two-fold: first, to understand the challenges they faced managing T2DM while experiencing binge eating—to add to nascent needs assessment literature [19, 21]—and second, to identify intervention adaptations to support users with T2DM. This manuscript offers new insights by engaging adults with T2DM immediately after binge-eating intervention and identifying promising adaptations that future work can design and test.

## Methods

### Participants and Procedures

This manuscript aligns with the Standards for Reporting Qualitative Research [36]. This analysis combines qualitative data from two completed trials of FoodSteps that recruited concurrently in 2024–2025; a figure outlining eligibility procedures by trial is in *Electronic Supplementary Material*. The first trial (“pilot trial”; MASKED) was a pilot project testing FoodSteps in adults with recurrent binge eating, T2DM, and food insecurity. These eligibility criteria were selected due to the high comorbidity of binge eating and T2DM [11], and between binge eating and food insecurity [37]. The second trial (“randomized trial”; MASKED) was a fully powered clinical trial of adults with recurrent binge eating and obesity; though T2DM was not an eligibility criterion, some participants in this trial had T2DM. All participants included in this analysis had a self-reported diagnosis of T2DM.

Recruitment for both studies prioritized enrolling a diverse sample based on gender, race, and ethnicity. For example, a subset of recruitment materials was developed to engage a socio-demographically diverse sample through targeted images and language. Recruitment streams included advertisements on public transit lines, flyers in clinics, and brochures distributed by our community partners [e.g., food pantries; 38]. Participants also were recruited online via social media and research registries [e.g., ResearchMatch; 39].

In both trials, interested individuals initiated enrollment by submitting an online screener which included questions from the Eating Disorder Diagnostic Scale [40]. At screening, potentially eligible participants were non-pregnant, English-speaking adults with self-reported recurrent binge eating (defined as ≥ 12 episodes in the past 3 months). Eligibility was subsequently confirmed in each trial via a baseline assessment, comprised of the Eating Disorder Examination interview [EDE; 41] and online surveys. Participants in the trials were eligible if they had ≥ 12 binge-eating episodes in the past 3 months, based on the EDE. Eligible individuals also were willing to use an app-based intervention and did not self-report a contraindicated clinical diagnosis (e.g., psychosis). Additional eligibility criteria differed by trial. In the pilot trial, participants had T2DM and food insecurity, and could have either objectively or subjectively large binge-eating episodes, based on the EDE. Episode size (i.e., amount of food consumed) is a clinical and diagnostic distinction used in eating disorder assessment [41]. In the randomized trial, participants had obesity (BMI ≥ 30 kg/m^2^) and ≥ 12 objectively large binge-eating episodes, per the EDE.

In both trials, eligible individuals who agreed to participate were given access to FoodSteps for four months with support from a coach; coach support was primarily delivered via messaging. Post-intervention, participants from both trials were invited to complete a research assessment that included an individual interview led by a trained assessor. To be included in this analysis, participants must have completed that interview. Randomized trial participants were compensated $60 for this research assessment and pilot trial participants were compensated $40.

All participants provided informed consent, either written (randomized trial) or verbal (pilot trial). The MASKED Institutional Review Board approved both studies.

### Measures

A T2DM diagnosis was self-reported at screening in both trials. Participants were asked if a clinician had ever diagnosed them with various medical and psychiatric conditions, including T2DM, and could indicate if their diagnosis was current or past. Only participants with current (not in remission) T2DM were included in this analysis. Gestational diabetes was not assessed.

At screening, demographics were self-reported, including height and weight, to calculate BMI. Participants also self-reported the name and dosage of any medication for binge eating, weight, or T2DM; so long as their dosage was stable for ≥ 30 days, this was not an exclusionary criterion. Household income and education were self-reported at baseline. Food security was assessed via the 6-item, 30-day Short Form Food Security Survey [42], administered at screening (pilot trial) or baseline (randomized trial). Scores ≥ 2 indicate low or very low food security. All surveys were administered via REDCap, a secure web-based platform [43], hosted at MASKED.

During the post-intervention assessments in both studies, a trained assessor administered a semi-structured interview (10–20 minutes) about participants’ experience in the intervention, ideas for new features, and health conditions and goals. Though each trial had a unique interview guide, analogous topics were explored. In the pilot trial, participants were explicitly asked about T2DM (*“What challenges do you face managing your diabetes? How could we improve FoodSteps to help you manage diabetes?”*). In the randomized trial, participants were asked about their health goals (*“Do you have other health goals, and if so, what are they? How could we improve FoodSteps to meet your other health-related goals?”*), and many brought up T2DM self-management without explicit prompting.

### Analyses

This study combined interview data from both trials to augment our sample and diversify the lived experiences we considered in answering our research questions. However, because randomized trial participants were not asked explicitly about T2DM, 12 individuals who were otherwise eligible to be included in this analysis, but did not mention T2DM, were excluded from the study. For reference, their demographics are in *Electronic Supplementary Material*.

Methods from thematic analysis [44] were used to analyze the interviews, which were automatically transcribed by Zoom. Trained team members reviewed the transcripts for accuracy and anonymized them. Transcripts were compiled in one document for analysis. It was infeasible to mask coders as to which data came from which trial, due to the trials’ distinct interview guides. The coding team included a clinical psychology doctoral student (XX), an undergraduate student (XX), and a bachelor’s-level research assistant (XX). Analyses occurred in fall 2025, during which coders met weekly to discuss themes. The coders began by reading the data in full, then inductively generated an initial codebook and individually applied it to the transcripts. After the coders met to discuss opportunities to improve the codebook, the lead author updated it, and each coder applied it to the transcripts. In November 2025, XX presented in-progress results to the authorship team, who discussed the codebook and themes. Afterward, XX updated and codified the codebook, and the coders applied it to the transcripts. Coding discrepancies were resolved by XX and the trials’ principal investigator (XX).

Toward the goal of self-reflexivity in qualitative research, the lead author discloses her identities as a White woman with advanced education whose graduate research focuses on optimizing binge-eating intervention for adults with chronic conditions. Other coders included an undergraduate student studying neuroscience/psychology who identifies as an Asian and Latina woman, and a post-baccalaureate researcher who identifies as a White man. No coders had current lived experience with binge eating or T2DM, which limited their perspectives during the coding process, but each coder had read literature on individuals’ lived experiences with T2DM and binge eating and/or collected interview data from individuals with T2DM and binge eating. In weekly meetings, coders reflected on their biases and took actions to ensure codes accurately reflected participants’ intended meaning (e.g., reading quotes aloud and discussing them).

## Results

Sample characteristics are in Table 1. In the combined sample, 74% of individuals self-identified as female, 24% as male, and 2% as another gender. On average, participants were 48 years old (SD = 11; range: 25 to 75). Fourteen percent self-identified as Hispanic; the others self-identified as non-Hispanic and Asian (5%), Black/African American (33%), more than one race (2%), Native American/Alaska Native (7%), and White (38%).

Participants cited challenges managing T2DM while experiencing binge eating and suggested intervention improvements. Their responses indicated considerations for intervention dissemination and service delivery. Our codebook, and representative quotes, are in Table 2.

### Theme 1: Disease Management Challenges

Most individuals described challenges due to T2DM or the intersections between T2DM and binge eating. Results in this theme indicate that adults with T2DM face internal and external/structural challenges that an adapted binge-eating intervention could address.

#### Knowledge challenges.

Participants said that they were unsure how to manage T2DM, especially regarding optimal diet and the impact of different foods on their blood glucose levels.

#### Cognitive/psychological challenges.

Participants described cognitive and psychological factors that undermined T2DM self-management, including forgetting to take their medications or check their blood sugar. Many said it was difficult to resist cravings for sugar, which consequently spiked their blood sugar. A few described potentially disordered eating behaviors in addition to binge eating, such as restrictive eating and skipping meals. Other barriers included not feeling motivated to change eating behaviors and disliking taking T2DM medications.

#### Physiological challenges.

Participants cited physiological challenges that complicated T2DM self-management, including difficulties being physically active and managing weight. Related medical concerns, especially high cholesterol and hypertension, were often described. Many participants shared general concerns about their hemoglobin A1c and blood glucose levels. Several also described how low blood glucose readings sometimes led to binge-eating episodes. These physiological challenges indicate areas where adapted supports are needed.

#### External/structural challenges.

Participants cited barriers to T2DM self-management, notably, low financial resources. Most who endorsed this theme came from the pilot trial, which required food insecurity. Participants said that the expense of healthy food and medication made T2DM self-management harder. Some described poor medical care, citing little clinical guidance. Others said they wanted, but did not often receive, social support from others.

### Theme 2: Intervention Adaptation Opportunities

Participants suggested adaptations to the intervention for T2DM, which were sorted into four subthemes, aligned with the challenges described in Theme 1. A majority preferred adaptations, whereas two participants did not perceive benefit in adapting the intervention for T2DM because their diabetes is well-controlled or because other T2DM-specific tools exist.

#### Psychoeducation and nutrition.

Many participants wanted psychoeducation about T2DM and the relationship between T2DM and binge eating, including self-management strategies for T2DM and binge eating, risk factors of T2DM progression, and tools like continuous glucose monitors. They suggested adding diabetes-friendly nutrition recommendations, such as meal plans, recipes, and ideas of foods to swap with low-carbohydrate alternatives.

#### Adaptations to address cognitive/psychological challenges.

To address motivation and memory challenges, participants suggested delivering nudges toward healthy behaviors and enabling personalized reminders for T2DM self-care tasks. Some suggested offering incentives for completing tasks like scheduling appointments or taking medications. Such features could reinforce keeping up with self-management and help sustain motivation for behavior change.

#### Adaptations to address physiological challenges.

Participants suggested adding content and features relevant to users with a range of physical abilities, such as physical activity ideas for individuals with physical limitations. Others wanted to track their blood glucose levels in the intervention, so that they could visualize how their eating behaviors impact their glucose and see their progress over time. Some wanted to track their steps and fitness goals in the app.

#### Adaptations to address external/structural challenges.

Participants suggested improving social support by adding a social forum to connect with other users and offering in-person meet-ups. Some expressed interest in sharing intervention data with their clinicians via their electronic health record. Some also suggested adding features to connect them with outside resources for social or medical support, such as local support groups and opportunities for free insulin. Other suggestions included covering T2DM medications and food deliveries as part of the intervention.

### Theme 3: Dissemination and Service Delivery Considerations

Duration of T2DM was identified as a subtheme, as participants said length of illness had implications for how and to whom intervention should be disseminated and delivered. They often described how their experience with T2DM had evolved, and several individuals with long-term T2DM said that although they needed less support with T2DM today, an adapted intervention would help individuals recently diagnosed. Regarding what might entice future users to enroll in intervention, several participants cited family relationships as motivation to control T2DM, while others said their fear of dying or acute health events motivated their behavior change. These motivators could be leveraged to engage adults with T2DM into binge-eating intervention.

## Discussion

### Principal Findings

This study used human-centered design methods to engage adults with T2DM who experience binge eating, and who had recently completed a digital binge-eating intervention, to learn about challenges they experience and opportunities to adapt the intervention for T2DM. Participants described experiencing challenges due to low knowledge (e.g., nutritional know-how), cognition/psychology (e.g., memory lapses), physiology (e.g., blood sugar management), and low resources (e.g., expensive medications)—indicating promising areas for intervention adaptation. This study found that adapted intervention could be most useful to those recently diagnosed with T2DM, posing implications for how and to whom the intervention could be disseminated. Taken together, findings add qualitative evidence supporting the need to adapt binge-eating intervention for T2DM. Results advance extant literature by examining individuals’ perspectives post-treatment and identifying intervention adaptations that future work can design.

### Individual-Level Challenges

The challenges participants described largely align with those faced by adults with T2DM in general. Low knowledge is a known barrier to dietary modifications [45], which are a central tenet of diabetes clinical care guidelines that individuals struggle to implement [46]. Participants recommended integrating nutritional recommendations, which is feasible as evidence-based guidelines are well-established [e.g., via the American Diabetes Association; 13]. Critically, for adults with T2DM who experience binge eating, these guidelines may not be wholly appropriate, as low-calorie, restrictive diets can exacerbate binge eating [47]. To this end, our intervention emphasizes *how* to eat (e.g., consistently), rather than *what* to eat. We suspect this support is key for individuals with T2DM and problematic eating behaviors, who are often advised to reduce weight without support implementing healthy eating behaviors. Still, this result indicates that future work could explore ways to integrate nutritional guidance in the intervention.

As unstable blood sugar impairs cognitive functioning [48], challenges related to memory and motivation are unsurprising; personalized reminders are a promising next step. Difficulties being physical active and managing blood sugar align with the experiences of adults with T2DM broadly [49, 50]. This study advances the literature via our finding that low glucose can instigate binge eating in adults with T2DM, a known relationship in type 1 diabetes [e.g., 51]; an adapted intervention can address this behavior. Also, 14% of our sample self-reported a current diagnosis of heart disease, and several referenced living with high cholesterol. The high treatment burden adults with T2DM already experience may be exacerbated by these diagnoses. Indeed, T2DM often involves stigma and self-blame [52]—factors that can worsen binge eating and T2DM self-management [19]. Psychoeducation on these constructs could be integrated into an adapted intervention. Intervention developers would benefit from accounting for the added burdens of comorbidities among adults with T2DM who are working on health behaviors.

### External/Structural Barriers to T2DM Self-Management

Participants cited structural factors that undermined T2DM self-care—namely, financial barriers, which can thwart self-management of T2DM [45] and binge eating [37]. Of note, 71% of the sample had food insecurity. This high proportion, also discussed in *Limitations*, reflects T2DM disparities across socioeconomic factors. Indeed, our results indicate that a binge-eating intervention adapted for T2DM must account for social determinants of health. Interventions delivered as part of governmental assistance programs could be a model to link individuals with resources and treatment, though research must explore the feasibility of this approach.

Participants described needing more medical support. Relatedly, whereas FoodSteps uses a guided self-help service model with low-intensity coaching, some wanted intervention data sent to their care team. This prospect requires careful implementation planning to avoid overtaxing clinicians, who often have limited time and environmental resources. This planning could be facilitated via human-centered design work with clinicians and medical systems.

Participants’ desire for added social support reflects how T2DM and binge eating are often isolating. Because FoodSteps is delivered by digital intervention, there is potential to link users together, though this raises workflow challenges for safe content moderation. While in-person opportunities are beyond the intervention’s scope, external referrals could be integrated.

### Considerations for Dissemination and Service Delivery

Though T2DM duration was not explicitly assessed, results indicate that an adapted intervention may be most useful to those recently diagnosed, when learning to manage T2DM is most salient. Referring patients to intervention at diagnosis aligns with the referral model of the National Diabetes Self-Management Education and Support program. Yet, T2DM self-care knowledge varies across individuals, and duration alone does not equate to higher knowledge [53]. Personalization by knowledge and health literacy could be an apt approach.

We identified positive (e.g., desire to be around for family) and negative motivators (e.g., fear of dying early) that could be used to engage individuals into intervention. Participants cited these factors as driving their behavior changes. Given the critical importance of self-management in T2DM, our findings indicate that future work should explore how to highlight these motivators in the intervention. As fear-based appeals may be less effective over time, the positive motivators may be most promising. Future work would benefit from assessing individuals’ intervention dissemination preferences to optimize engagement efforts [e.g., 54].

### Clinical Implications

This manuscript used qualitative methods to examine an under-addressed area of diabetes care, with implications for clinicians. Unhealthy eating behaviors such as binge eating can thwart weight management, which is core to the current paradigm of T2DM care. While care often centers *what* to eat, less attention is paid to healthy eating behaviors (e.g., eating regularly) and common problems patients may experience (e.g., skipping meals, binge eating). Primary care clinicians would benefit from increased awareness of how eating behaviors can undermine self-management practices and outcomes, as well as when and how to screen for eating problems and refer patients to specialty care. This could be facilitated via strategies such as live didactics or toolkits, but to promote uptake, these resources must be designed to align with clinicians’ preferences, levels of knowledge, and openness to integrating this focus into care.

Our results indicate that patients’ experiences of care and clinical outcomes could be improved by addressing eating problems in diabetes care. Despite the potential impact of doing so, this recommendation is challenging because diabetes care clinicians often have low capacity in appointments, and insufficient training in behavior change, to offer ongoing behavioral intervention [55]. Interventions with a self-contained service model that do not require clinicians’ time may be optimal. Also, our findings and others [e.g., 19] indicate a need for treatment guidelines to address eating behaviors in diabetes care to support clinicians who encounter patients with eating problems in routine clinical practice. Integrated care, with coordination across physical and mental health clinicians, may be promising to improve care for patients with T2DM and eating problems.

Clinicians who treat patients with T2DM could routinely assess eating behaviors. Dialogue tools for diabetes clinicians to talk with patients about eating behaviors are in development [27], though assessments that can be completed outside appointments are also important to explore. Short screening tools such as the Diabetes Eating Problem Scale-10 [26] may be an efficient way to identify patients who would benefit from eating-related resources.

### Limitations

Study strengths included a socio-demographically diverse sample of adults with T2DM who experience binge eating; however, limitations must be noted. Participants were aggregated from two trials with distinct eligibility criteria, such that the included individuals differed in several ways. Pilot trial participants could be eligible with recurrent subjective or objective binge eating, whereas randomized trial participants needed recurrent objective binge eating; pilot trial participants also had low or very low food security. In combining these data, we aimed to capture a greater diversity of lived experiences, but results may not generalize to individuals with solely objective binge eating or higher food security levels. Data were elicited using different questions in each trial. Although participants across trials endorsed similar codes and themes, study rigor would have been strengthened by using identical questions in each trial. Only five participants (12%) were using insulin, meaning our results do not capture the perspectives of many individuals with uncontrolled diabetes; none endorsed insulin restriction to manage weight. Participants also did not describe how their medications impacted diabetes and binge eating. Future work is needed to map how medications differentially impact appetite and binge eating, to inform intervention content and design. Our sample also was majority female (74%), a persistent challenge in binge-eating research [56]. Future work should prioritize recruiting more men to improve generalizability. Finally, a unique contribution of our analysis is that all participants had recently completed a binge-eating intervention, enabling us to report individuals’ perspectives on their treatment experiences. However, the challenges these individuals described may differ from the challenges faced by individuals with T2DM who have never received intervention. Follow-up design work should engage these individuals as well.

## Conclusion

This study adds evidence supporting the need to develop adapted binge-eating care for adults with T2DM. Findings demonstrate areas where binge-eating intervention can be adapted to improve relevance for adults with T2DM. These design ideas offer a foundation for future work, and can now be validated with target end-users, and eventually, deployed in an adapted intervention. Results reinforce the importance of identifying and intervening on impairing eating behaviors in routine diabetes care to better support adults with T2DM.

## Supplementary Material

Supplementary Files

This is a list of supplementary files associated with this preprint. Click to download.

- Supplementalmaterial.docx

## Figures and Tables

**Table 1:** Sample characteristics, aggregated by trial.

Construct	Combined sample (*n* = 42)	Randomized trial participants (*n* = 16)	Pilot trial participants (*n* = 26)
	Mean (SD)		
**Age (years)**	48.4 (11.3)	51.2 (11.8)	46.7 (10.9)
**Annual household income (USD)**	$52,400 ($51,200)	$80,200 ($70,700)	$35,700 ($23,900)
**Baseline body mass index (kg/m** ^**2**^ **)**	38.8 (8.36)	35.9 (5.99)	40.6 (9.19)
**Binge-eating episodes in the past 3 months**			
Total episodes	41.9 (22.4)	40.5 (23.4)	42.7 (22.1)
Objectively large episodes	29.4 (24.3)	39.4 (24.5)	23.2 (22.4)
Subjectively large episodes	12.5 (21.5)	1.13 (2.50)	19.5 (24.9)
**Gender**	**n (%)**		
Female	31 (73.8%)	11 (68.8%)	20 (76.9%)
Male	10 (23.8%)	5 (31.3%)	5 (19.2%)
Other	1 (2.4%)	0 (0%)	1 (3.8%)
**Past-month food insecurity**	30 (71.4%)	4 (25.0%)	26 (100%)
**Bachelor’s degree or higher**	19 (45.2%)	9 (56.3%)	10 (38.5%)
**On medication for diabetes, weight, or binge eating**			
Insulin therapy (with or without additional medications)	5 (11.9%)	1 (6.3%)	4 (15.4%)
Non-insulin medication(s) only	17 (40.5%)	7 (43.8%)	10 (38.5%)
**Hispanic/Latino**	6 (14.3%)	3 (18.8%)	3 (11.5%)
**Race**			
Asian	2 (4.8%)	1 (6.3%)	1 (3.8%)
Black or African American	14 (33.3%)	6 (37.5%)	8 (30.8%)
More than one race	1 (2.4%)	1 (6.3%)	0 (0%)
Native American/Alaska Native	4 (9.5%)	2 (12.5%)	2 (7.7%)
White	21 (50.0%)	6 (37.5%)	15 (57.7%)
**Current chronic condition diagnoses**			
Heart disease	6 (14.3%)	3 (18.8%)	3 (11.5%)
Kidney disease	2 (4.8%)	0 (0%)	2 (7.7%)
Liver disease	3 (7.1%)	0 (0%)	3 (11.5%)
Stroke (past or present)	2 (4.8%)	0 (0%)	2 (7.7%)
Chronic pain	16 (38.1%)	7 (43.8%)	9 (34.6%)

**Table 2:** Themes, codes, and representative questions.

Theme	Subtheme	Codes	Example Quote(s)
**Disease Management Challenges**	Knowledge challenges	Lack of T2DM and/or nutritional knowledge	“I personally don’t know anything about diabetes, and like what you’re supposed to eat or not eat.” (Native American female, PT)
	Cognitive/psychological challenges	Remembering T2DM management tasks Resisting sugar Other eating problems Other mental barriers	“I also have a mixed history of binge eating and restricted eating. So, I’ve worked really hard over the past year or so on the restricted eating because of my diabetes.” (White female, RT) “Moderation, that’s the number one thing …Not over-enjoying it to where, ‘Oh, my blood sugar’s high!'’“ (Black male, PT)
	Physiological challenges	Difficulties being active Difficulties managing weight Medical comorbidities Difficulties with blood sugar	“Now they got it where it [my blood sugar] doesn’t raise real high. Now, my problem is, it’s going way too low, and I’m like, ‘Ha ha it’s too low, I get cheesecake,’ and there it goes, way up high.” (Hispanic female, RT)
	External/structural challenges	Lack of resources Low social support Insufficient medical care	“Sometimes, there is no food. You’re sitting here eating ramen. Super cheap, and it makes my diabetes go up.” (White female, PT) “Sometimes I need to talk to somebody that is going through the same thing I’m going through.” (Hispanic female, RT)
**Intervention Adaptation Opportunities**	Psychoeducation and nutrition	Psychoeducation Nutrition information	“A binge for any of us is serious enough. But a binge for a diabetic causes other issues. Being aware of that…how serious it is, and how simple it can be to manage it differently.” (White female, PT)
	Adaptations for cognitive/psychological challenges	Nudges/reminders Incentives to motivate T2DM management	“For instance, Metformin, I take it once a day, so I have an alarm when I have to take all my medication. But if that can be in FoodSteps, that’s great.” (Asian female, RT) “There could be a point incentive if you go see your PCP.” (Native American female, PT)
	Adaptations for physiological challenges	Adaptive physical activity support Blood sugar tracker	“Maybe a variety of ways to do exercise. People always say, ‘Well, go out there and walk.’ One thing is, it hurts; everything, it hurts.” (White female, PT)
	Adaptations for external/structural challenges	Discussion forum In-person social support Food delivery Community resources for social/medical support Send data to doctor Medication subsidies	“It would be cool to have that social interaction, but not from professional people…Another thing would be a place where you could go to like share recipes.” (Hispanic female, RT) “We could have met up and done like a half-hour walk, and then like a 10–20 min info session on something.” (Black female, PT) “If you could link the program to the doctor, or offer that, that would be fantastic.” (White female, PT)
	Adaptations are not necessary	Does not need T2DM adaptations	“No, because I feel that’s [diabetes] a personal thing, because I don’t take any type of medication.” (Black male, RT)
**Considerations for Dissemination and Service Delivery**	Duration of T2DM	Duration of T2DM	“How to get started when you’re brand new [with diabetes], and you don’t know what you’re doing. That would be a great.” (Native American female, PT) “I’ve been working on it [T2DM] for a while, so I sort of know what to do. But if someone is new with diabetes, then it might help them if they don’t know what to do.” (White female, RT)
	Motivators	Motivated by goals/values Motivated by fear of negative outcomes	“It was at a point where if it got any worse, I might not be able to come back. So, I made those changes, and ever since then it’s just been improving, my blood sugars.” (Hispanic female, PT) “I just want to be as healthy as possible, so that I could show up where I need to show up for my family. And so those were the things that were driving me.” (Black female, RT)

*Note*. T2DM = Type 2 diabetes mellitus; PT = pilot trial; RT = randomized trial; PCP = primary care provider.

## Data Availability

Deidentified data may be made available upon reasonable request to the principal investigator.
